# Denosumab in Giant Cell Tumor of Bone: Current Status and Pitfalls

**DOI:** 10.3389/fonc.2020.580605

**Published:** 2020-10-02

**Authors:** Hengyuan Li, Junjie Gao, Youshui Gao, Nong Lin, Minghao Zheng, Zhaoming Ye

**Affiliations:** ^1^Department of Orthopedics, Centre for Orthopedic Research, School of Medicine, Orthopedics Research Institute, Second Affiliated Hospital, Zhejiang University, Hangzhou, China; ^2^Centre for Orthopaedic Research, School of Surgery, The University of Western Australia, Nedlands, WA, Australia; ^3^Department of Orthopaedic Surgery, Shanghai Jiao Tong University Affiliated Sixth People's Hospital, Shanghai, China

**Keywords:** giant cell tumor of bone, denosumab, local recurrence risk, malignant transformation, H3F3A G34W

## Abstract

Denosumab is a monoclonal antibody against RANK ligand for treatment of giant cell tumor of bone (GCTB). Clinical trials and case series have demonstrated that denosumab is relevant to beneficial tumor response and surgical down-staging in patients of GCTB. However, these trials or case series have limitations with a short follow-up. Recent increasing studies revealed that denosumab probably increased the local recurrence risk in patients treated with curettage. This may be caused by the thicken bone margin of tumor that trapped tumor cells from curettage. The direct bone formation by tumor cells in the margin after denosumab treatment also contributed to the local recurrence. *in vitro* studies showed denosumab resulted in a cytostatic instead of a true cytotoxic response on neoplastic stromal cells. More importantly, denosumab-treated GCTB exhibited morphologic overlap with malignancy, and a growing number of patients of malignant transformation of GCTB during denosumab treatment have been reported. The optimal duration, long term safety, maintenance dose, and optimum indications remain to be elucidated. With these concerns in mind, this review warns that the denosumab therapy of GCTB should be applied with caution.

## Introduction

Giant cell tumor of bone (GCTB) is a primary intermediate bone tumor with a local aggressive behavior (1). It accounts for ~4–5% of all primary bone tumors, with peak incidence in the second to fourth decades of life (2, 3). GCTB has a rare tendency to metastasize, but there is a soaring risk of pulmonary metastasis in those advanced or recurrent patients (4).

The main treatment modality of GCTB is surgery, which includes en bloc resection and extensive curettage with adjuvants. Ideally, extensive curettage combined with high-speed burring and local adjuvants should be the first choice and achieves salvage of joint adjacent to the tumor, although it has a higher recurrence rate. En-bloc resection is recommended as for the tumors with far-ranging bone destruction and soft tissue extension. It minimizes the risk of local recurrence but correlates with a higher rate of surgical complications and functional impairment. The local recurrence rate of GCTB ranges from 27 to 65% for curettage alone, from 12 to 27% for curettage combined with adjuvants and from 0 to 12% for en-bloc resection (5–8).

The discovery of the crucial role of RANK/RANKL pathway in the pathogenesis of GCTB has given rise to the development of denosumab, a fully human monoclonal antibody against RANKL. As neoadjuvant therapy for advanced GCTB which is unresectable or where surgical resection probably results in severe morbidity, denosumab is the only medicine approved by US Food and Drug Administration (FDA) and the European Medicines Agency (9). Numerous clinical trials have shown denosumab correlates with beneficial tumor response (10–12). However, recent studies revealing a higher rate of recurrence and patients of sarcomatous transformation of GCTB after denosumab therapy are reported increasingly (13–15). We were the first who reported that RANK/RANKL pathway was essential in the pathogenesis of GCTB (16), herein, it is time to put vital information together to create a full-scale review for denosumab therapy.

## Histopathology and Genetics of GCTB

The better understanding of histopathology and molecular biology has led to the progress of denosumab for GCTB (17). Grossly, GCTB is well-vascularized and friable in texture with a dark brown-to-reddish appearance. Cystic degeneration, hemosiderin deposition, and hemorrhage can be seen, especially common in larger tumors. Microscopically, GCTB are typically comprised of RANK-positive circular mononuclear cells, “reactive” rich RANK-positive multinucleated giant cells, “neoplastic” densely cellular spindled RANKL-positive stromal-like tumor cells, areas of sparse osteoid matrix and woven bone (16, 18). Clues gleaned from studies corroborate that stromal cells, representing an immature osteoblast phenotype which originates from mesenchymal stem cells, are the true neoplastic part of GCTB because of their capacity to grow in cell-culture setting from generation to generation and form GCTB in mice (19–21).

Concerning functional biology, although a variety of cytokines such as SDF-1, MCP-1, VEGF, or M-CSF are involved, RANKL seems to be a core factor in the pathogenesis of GCTB (22). Overexpression of RANKL by stromal cells not only promotes recruiting monocyte precursors but also assists to form multinucleated osteoclast-like giant cells (23). Compared with RANK, denosumab has higher specificity and affinity to RANKL. As a result, denosumab could interrupt the RANK-RANKL binding which is necessary for osteoclast formation, leading to the elimination of osteoclast-like giant cells.

Karyotype analyses of tumor specimens displayed that chromosomal aberrations, including deletion, insertion, translocation, and other numerical or structural chromosomal rearrangement, are the common feature of GCTB (24, 25). Telomeric associations, where two diverse chromosome arms fuse together at ends, is the most prevailing cytogenetic finding. These associations are present in at least 70% of patients (24) and also found in isolated stromal cells (26). Telomeric associations are relevant to aberrations of clonal chromosome and reduction of telomere length, suggesting that telomeric instability is probably considered a vital core factor in the pathogenesis of GCTB (27, 28). However, no definite correlation between these cytogenetic abnormalities and established clinical grading systems or unfavorable clinical prognosis has been established (24, 25).

Recently, a distinct driver mutation H3F3A encoding the histone variant H3.3 has been identified in GCTB, with G34W accounting for the vast majority and G34L for a small minority (29, 30). This mutation is found in virtually all GCTBs, ranging from 92.0 to 97.8% (30, 31). It is confined to stromal cells and not detected in osteoclasts or precursors. IHC expression of G34W is more specific, sensitive and valuable for differential diagnosis from other histologically ambiguous giant cell-rich lesions including chondroblastoma, malignant giant cell-rich osteosarcoma and aneurysmal bone cyst. Even in metastatic, recurrent, secondary malignant and post-denosumab GCTBs, H3F3A G34W mutation and its IHC expression are maintained (32). Knockdown of this mutation counteracts the neoplastic phenotype, implying that H3F3A-G34W is sufficient to drive tumorigenesis of GCTB (33). Although mechanisms by which this mutation might drive tumorigenesis are still not fully understood, H3F3A-G34W presents a promising target for novel GCTB therapy.

## Outcomes of Clinical Trials and Case Series on GCTB After Denosumab Treatment

The discovery of giant cells in GCTB expressing RANKL (16, 18) has led to the clinical application of denosumab in treatment of surgical undecidable tumor. Consequently, a first open-label phase II proof-of-concept study was conducted in Thomas et al. (10), who reported that 30 of 35 (86%) of patients had a tumor response to denosumab treatment, defined as at least 90% elimination of giant cells on histological evaluation or lack of radiological progression of the target lesion. However, this study contains a small sample size of selected population and only a small part of patients received intralesional curettage after denosumab. A second phase II study evaluating denosumab in 282 patients confirmed the efficacy and safety of denosumab in GCTB (11). One hundred and sixty three of 169 (96%) patients with surgically unsalvageable disease (cohort 1) had no disease progression after denosumab treatment. Of 100 patients with planned surgery (cohort 2), 16 of 26 patients underwent less morbid surgery than originally schemed, and 74% of patients had no surgery. The authors concluded that denosumab was effective and can prevent or reduce the morbidity of the planned surgery. However, the follow-up of roughly 1 year (median, 13 months for cohort 1, 9.2 months for cohort 2) is too short to reliably prove efficacy and safety in complicated cases of GCTB. It is noteworthy that sponsor Amgen was strongly involved in study design, assessment, and interpretation of data with potential bias. Another clinical trial of open-label phase II accessed the reduction in surgical invasiveness after denosumab therapy (34). Forty-eight percentage of 222 patients had no surgery or a less morbid procedure. Of the 116 patients who underwent surgery, 17 patients (15%) developed local recurrence. The median duration of follow-up for patients received surgery was 13 months. The median duration to recurrence was 13.6 months, postoperatively. Longitudinal institutional cases and collaborative group studies showed that local recurrence was inclined to occur principally within the first 12–18 months after surgery (35–37). Notably, the median follow-up of this study was insufficient, even shorter than that in local recurrence cases. Recently, Chawla et al. (12) presented the long-term follow-up results of their phase 2 trial of denosumab. They expanded the trial from 282 patients in the interim analysis (11) to 532 patients, and thereby completed the largest clinical trial to date on GCTB using denosumab. The authors showed that only 11% of patients with unresectable disease had progression after 65.8 medium months follow-up and 92% of the resectable patients had no surgery for GCTB during the first 6 months. However, 31 (34%) of 90 patients with resectable GCTB had tumor recurrence after curettage. Notably, 20 (4%) patients with a possible diagnosis of malignancy were identified. Although three-quarters of these patients were excluded from sarcomatous transformation since authors believed they had been misdiagnosed at baseline, the dubious association between denosumab and malignant transformation was still the event of great interest. To sum up, although these clinical trials presented promising results of denosumab in GCTB, it should be interpreted with caution by reason of short follow-ups, high risk of recurrence, potential malignant transformation and possible interference of funder.

## Denosumab May Increase the Risk of Local Recurrence in Patients of GCTB Treated With Curettage

GCTB usually leads to expanded and thinned cortical bone at diagnosis, which could be prone to perforation with minimal pressure at surgical resection or curettage. After an average of 3–4 months of denosumab, a reduction in tumor size and the new ossified tumor matrix can be seen (38). It seems that neoadjuvant denosumab could promote en bloc resection and intralesional curettage via developing a calcified rim around the whole tumor and its soft tissue component. However, the local recurrence of curettage with neoadjuvant denosumab fails to be improved, even getting worse. In a prospective non-randomized study of patients who received denosumab for 6–11 months before intralesional curettage surgery, Traub et al. (39) reported a local recurrence rate of 17% (3/18) with the median follow-up of 30 months (range, 20–45 months). The local recurrence rate was comparable with those in other studies without denosumab treatment, indicating that denosumab may not improve local control of GCTB after curettage.

Errani et al. (13) reported a higher local recurrence rate in the cohort at a median follow-up of 42.1 months (range, 37.4–50.8 months). The local recurrence rate was as high as 60% (15/25) of patients with denosumab and curettage compared with 16% (36/222) of patients with isolated curettage. Denosumab was the only independent element correlated with a poor prognosis in view of recurrence-free survival. Although causation may not be evaluated by reason of substantial differences in the cohorts, such a high local recurrence rate dampens the enthusiasm and pushes us to reconsider the role of denosumab in curettage.

Other studies confirmed these results (Table 1). Agarwal et al. (15) conducted a case-matched comparison study to rule out some confusion involving denosumab. They reported that 44% (11/25) of patients in the denosumab and curettage group had local recurrences in comparison with 21% (7/34) in the control group without denosumab, although it was not statistically significant. They recommended to reduce the doses of denosumab before curettage to just adequate for bone formation and believed that it was crucial to curette and burr up to margins on initial images with the help of intraoperative C-am.

**Table 1:** Summary of published studies reporting higher local recurrence rate of GCTB after neoadjuvant denosumab following curettage.

**No. of study/references**	**Patients^†^**	**Follow-up (months)**	**Local recurrence in denosumab plus curettage**	**Local recurrence in curettage alone**	**Doses or months of denosumab**	**Time of local recurrence (months)**
Errani et al. (13)	25	Median 42	60% (15/25)	16% (36/222)	NR	Median 15
Agarwal et al. (15)	25	Median 27	44% (11/25)	21% (7/34)	Doses, Median 6.8	NR
Scoccianti et al. (40)	12	Median 39	41.6% (5/12)	11.1% (1/9)	NR	Median 23
Puri et al. (41)	25	Median 30	44% (11/25)	NR	Doses, Mean 5	Mean 16
Medellin et al. (42)	4	Median 75	100% (4/4)	39% (9/23)	Months, Mean 8.9	NR
Chinder et al. (43)	42	Mean 35	42.8% (18/42)	18.5% (15/81)	Months, Mean 2.9	Mean 12.9

*NR, not reported*.

† *Numbers of patients in neoadjuvant denosumab plus curettage*.

We have speculated that denosumab treatment results in less removal of osseous tumor matrix and thus thickened tumor margin wall. As a result, the outline the true scope of tumors is no longer exist. In addition, tumor cells may be entrapped within the thickened new bone. In support of this, Muller et al. (44) revealed that viable tumor cells persisted in the new-formed bone induced by denosumab by means of histologic analysis. They suggested that the surgical technique of curettage had to be more aggressive to reduce higher local recurrence rate. Cryotherapy was recommended because the penetration depth in the adjacent bone was likely the best. However, in a retrospective study of patients with GCTB who received curettage and cryotherapy, Scoccianti et al. (40) showed a recurrence rate of 41.7% in 12 patients received denosumab in comparison to 11.1% in 9 patients in the surgery-only group, although it was not statistically significant. Puri et al. (41) revealed a recurrence rate of 29% in 41 patients who received preoperative denosumab with a mean follow-up of 34 months. Local recurrence occurred in 44% (11/25) of patients who had curettage, much higher than the resection group (1/16, 6%). Medellin et al. (42) conducted a study of 107 patients with pathological fracture due to GCTB, aiming to explore the prognostic factors for local recurrence. All patients who received denosumab combined with curettage, albeit only 4 cases, developed local recurrence. The authors found that denosumab was the only independent factor relevant to local recurrence by multivariate analysis, although the other two factors, the initial type of treatment and the location of the tumor, played roles on univariate analysis. Chinder et al. (43) conducted a study of 123 patients to evaluate the local recurrence of neoadjuvant denosumab following extensive curettage. This study was well-matched for the site of tumor and the type of surgery. The local recurrence rate in denosumab group is 42.8% (18/42), significantly higher than that of 18.5% (15/81) in curettage alone group. On multivariate analysis, neoadjuvant denosumab was the only independent risk factor for local recurrence following curettage. Recently, Tsukamoto et al. (45) performed a systematic analysis of seven studies with 619 patients and showed that the proportion of patients with local recurrence ranged from 20 to 100% in the curettage with preoperative denosumab compared with 0–50% in the curettage-alone group. The authors believed denosumab may be associated with an increase in local recurrence although the evidence was weak due to lack of randomized studies and indication bias. In another meta-analysis covering 10 studies with 1,082 cases, Chen et al. (46) found that denosumab therapy was correlated with higher local recurrence rate and inferior 5-year recurrence-free survival.

Several *in vitro* studies focused on the osteoclastogenic properties and viability of neoplastic stromal cells following denosumab therapy. Mak et al. (47) revealed that proliferation of stromal cells was only diminished by denosumab; once the micro-environment was free of the RANKL antibody, stromal cells remained proliferative, albeit to a lesser degree (~50% slower). Shibuya et al. (48) isolated three types of cells from GCTB patients and displayed that denosumab inhibited osteoclast differentiation and bone resorption but had no inhibitory effects on survival of osteoclasts or proliferation of stromal cells. Another study in comparison with zoledronic acid also showed that denosumab lacked anti-tumor effect against neoplastic stromal cells and raised a concern that local recurrence may occur in case of drug withdrawal (49).

The high rate of local recurrence after denosumab treatment may be interpreted as follows. Firstly, denosumab only targets multinucleated osteoclastic cells, rather than stromal cells. The neoplastic cells of GCTB still exist and has partial functions after denosumab therapy. Secondly, the typical soft tissue tumor of GCTB is altered into a gritty fibro-osseous matrix by denosumab treatment. That results in the tumor less defined from the ambient normal bone macroscopically and microscopically, making the decision of the extent of surgical curettage more intractable. In addition, denosumab also gives rise to thickening of the subchondral and cortical bone. As the circumferential bony layer thickens, it is probably that tumor cells get trapped within the new bone. Curettage is restricted by a thick bony shell to burr against. That most likely leads to tumor being inadvertently left behand after curettage and contributes to the local recurrence.

Some studies suggested that one of methods to promote identification of the boundary of tumor area and tissue response induced by denosumab is the usage of intra-operative fluoroscopy (15, 39). Using a C-arm intraoperatively, Agarwal et al. (15) reported a decreased recurrence rate from 57% (8/4) to 26% (6/23). They highlighted the importance of curettage to margins on pretreatment imaging. A shorter duration and lower doses of neoadjuvant therapy were also presented to reduce the risk of recurrence (9, 15, 38, 50). Maximum 3–4 months was deemed as the optimum time frame before intralesional surgery, considering that 6 months made the possibility of recurrence rate higher by getting the bony shell thicker and trapping more tumor cells. Hindiskere et al. (51) found that there were no significant differences between short-course (there or fewer doses) and long-course (more than three doses) groups of preoperative denosumab in terms of clinical scores, histological and radiological response or local recurrence survivorship. Short-course could reduce costs and complications linked with long-course therapy. However, Tsukamoto et al. (45) found that the preoperative denosumab duration did not seem to be associated with local recurrence after curettage by a systematic analysis of previous studies. Currently, the Japan Clinical Oncology Group is undertaking a randomized Phase III trial with 106 patients to ascertain the effect of preoperative denosumab on recurrence following curettage (52). More larger, multicenter and ideally prospective trials are warranted to reach concrete conclusions.

## Denosumab May Arouse the Malignant Transformation of GCTB

The potential for malignant transformation of GCTB is a rare but crucial consideration. Malignant GCTB was first described 80 years ago (53). Histologically, it could be a fibrosarcoma, osteosarcoma or undifferentiated high-grade pleomorphic sarcoma (54). Malignant GCTB is considered as either primary or secondary and comprises about 4% of all GCTB (55). Primary cases are adjacent to benign GCTB and secondary ones develop from previously treated GCTB. Most malignant GCTB is secondary with a poor prognosis. Five-year disease-free survival of secondary GCTB was 32% (56). Multiple local recurrences and previous radiation therapy have been proposed as predisposing factors for malignant transformation (54, 55). Recently, cases of malignant transformation of GCTB during denosumab treatment have been reported growingly (Table 2). Thomas et al. (10) reported the first cases in the initial phase II study. New sarcomas occurred in two patients; one developed a high-grade sarcoma in the upper extremity during denosumab therapy and the other had a malignant GCTB with lung metastases after ceasing denosumab. In the second phase II study (11), new primary sarcomas occurred in two patients: one was deemed as a malignant transformation while the other was suspected to be present at baseline retrospectively. In the study of 222 patients of GCTB who received denosumab therapy, Rutkowski et al. (34) reported that four patients developed malignant transformation. Two of them were considered as radiation-associated sarcomatous transformation and the diagnosis of the other two was missed by sampling error at initial core biopsy. Broehm et al. (57) showed that two patents receiving denosumab developed malignant transformation of GCTB to osteosarcoma. Before sarcomatous transformation occurred, both patients responded to denosumab well. Aponte-Tinao et al. (14) reported a high-grade sarcoma arising in a woman aged 20 with a recurrent GCTB while receiving denosumab. Park et al. (58) presented a patient with a large GCTB of pelvis who received denosumab therapy. Seven months after surgical excision, the patient developed an osteosarcoma in the same site, along with pulmonary metastasis. Tsukamoto et al. (59) demonstrated a 25-year-old woman with recurrent GCTB in her left ischium developed a high grade osteosarcoma. She received denosumab therapy for 6 months. Agarwal et al. (15) reported that a patient with a proximal humerus GCTB developed osteosarcoma after 8 months of curettage, and then developed pulmonary metastases and died of disease. Chen et al. (60) reported that a male patient aged 43 with sacral GCTB developed secondary malignancy by postoperative pathological examination. This patient received 4 doses of denosumab before surgery and responded well. However, the tumor progressed rapidly and led to his death after 6 months. In the largest clinical trial to date on GCTB using denosumab, Chawla et al. (12) reported that 20 (4%) of 532 patients developed new malignancies. Fifteen cases were excluded from malignant transformation since authors believed they had been misdiagnosed at baseline, the other five were determined to be secondary malignant GCTB or sarcomatous transformation.

**Table 2:** Summary of published studies reporting malignant transformation of GCTB after denosumab therapy.

**References**	**Year**	**Patients**	**Age/gender**	**Location**	**Time interval^†^ (months)**	**Time of denosumab (months)**	**Histology of sarcoma**	**Treatment**	**Outcome**
Thomas et al. (10)	2010	2	NR	Upper extremity	Range 3–7	Range 3–7	HGS	Resection	NR
			NR	Lung	Range 11–15	Range 3–7	MGCT	Resection	DOD
Chawla et al. (11)	2013	2	NR	NR	NR	NR	NR	NR	NR
Rutkowski et al. (34)	2015	2	NR	Pelvis, sacrum	8.6	8.6	NR	NR	NR
Broehm et al. (53)	2015	2	49/M	Ischium, pubis	31	30	OS	Chemotherapy	M, AWD
			46/M	Distal femur	6	6	OS	Wide resection + chemotherapy	DOD
Aponte-Tinao et al. (14)	2015	1	20/F	Proximal tibia	13	13	HGPS	Amputation	DF
Park et al. (54)	2016	1	28/F	Pelvis	34	20	OS	Hemipelvectomy + Chemotherapy	M, DOD
Tsukamoto et al. (55)	2017	1	29/F	Ischium	6	6	OS	Chemotherapy	M, DOD
Agarwal et al. (15)	2018	1	27/M	Proximal humerus	14	6	OS	Chemotherapy	M, DOD
Chen et al. (56)	2018	1	43/M	sacrum	2	3.5	MGCT	NR	DOD
Chawla et al. (12)	2019	5	NR	NR	NR	NR	HGS, MGCT	NR	NR

*NR, not reported; HGS, high-grade sarcoma; MGCT, malignant; GCT, giant cell tumor of bone; DOD, death of disease; HGPS, high-grade pleomorphic sarcoma; DF, disease free; OS, osteosarcoma; M, metastases; AWD, alive with disease*.

† *Time interval from start of denosumab therapy to diagnosis of malignant transformation of GCTB*.

In these above-mentioned cases, all patients responded to denosumab until malignant transformation occurred. To the best of our knowledge, up to now, about 18 cases of malignant transformation of GCTB during denosumab treatment have been reported. Obviously, the cases are rare, and thus it cannot be concluded that there is a definite direct cause-and-effect correlation between denosumab and malignant transformation. Since radiotherapy was thought to be closely correlated with malignant transformation of GCTB (55), we could make a comparison of time interval from the start of therapy to diagnosis of malignant transformation between denosumab and radiotherapy. In the above-mentioned cases, the interval from start of denosumab to diagnosis of the malignancy was 0.2–2.8 years with the mean of 1.1 years. Bertoni et al. (61) reported that six patients of GCTB who received radiotherapy developed malignant transformation. The interval from start of radiotherapy to diagnosis of the malignancy was 1.7–15 years with the mean of 8 years. Apparently, the interval of denosumab is much shorter than that of radiotherapy.

The potential mechanisms of sarcomatous transformations of GCTB following denosumab therapy are probably associated with its actions against RANKL (59). Although the exact molecular basis is poorly defined, three possible hypotheses are proposed. Firstly, it has been questioned if denosumab affects immunity and inflammation, since RANKL plays critical roles in lymphocyte development and lymph-node organogenesis (62–64). The inhibition of RANKL could increase the risk of new malignancies as a result of immunosuppression. Secondly, in osteosarcoma cells, RANKL expression increases the level of nuclear factor IB (NKIB) (65), a transcription factor which exhibits tumor suppressive functions in many malignancies via down-regulating susceptibility to nuclear oncogenes (66). Thus, restraint of RANKL could lead to osteosarcoma carcinogenesis by raising susceptibility to nuclear oncogenes. Thirdly, RANKL upregulates the level of Sema3A gene in osteosarcoma (65), and deletion of this gene could lead to aberrant cartilage and bone growth (67, 68). As a result, it is possible that restraint of RANKL by denosumab induces aberrant osteoblasts differentiation and osteosarcoma tumorigenesis via Sema3A.

## Morphological, Immunohitochemical and Molecular Changes of GCTB After Denosumab Therapy

**Figure 1:**
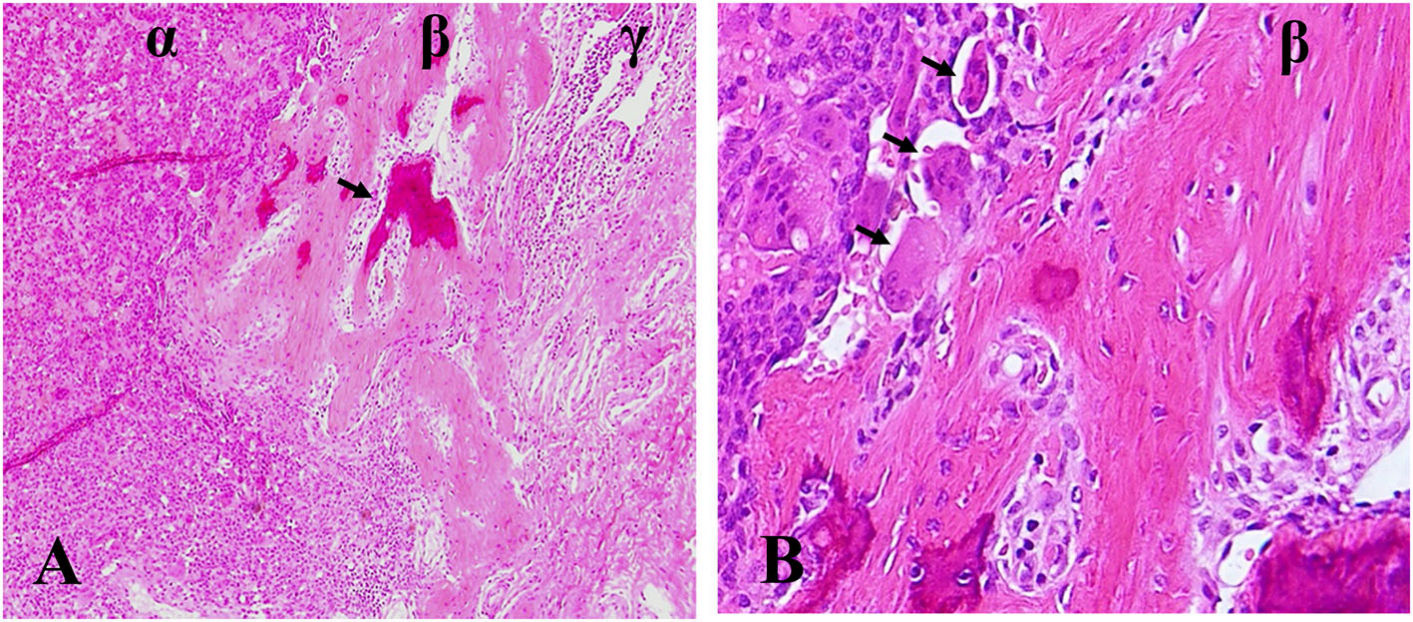
Ectopic osteoid formation in lung tissue of denosumab-treated patient following GCTB metastasis. **(A)** α zone, GCTB, β zone, fibroblastic osteoid-forming, γ zone, pulmonary tissue, arrow, osteoid. **(B)** β zone, fibroblastic osteoid-forming, arrow, osteoclastic cells. Hematoxylin and eosin statin, magnifications ×100 **(A)**, ×400 **(B)**.

The histologic changes of GCTB after denosumab treatment are variable and consist of depletion of giant cells, a reduction of neoplastic stromal cells, and incremental fibro-osseous tissue and/or new woven bone. We even found the ectopic osteoid formation in lung tissue of denosumab-treated patient following GCTB metastasis (Figure 1). These changes are so dramatic that the lesions treated by denosumab do not have any resemblance to the original ones and it is probably confused with malignant tumors. Wojcik et al. (69) examined 9 cases of denosumab-treated GCTB and demonstrated that tumor samples zexhibited morphologic overlap with malignancy. Early lesions were highly cellular and the combination of cellularity, atypia, and haphazard bone deposition were reminiscent of high-grade osteosarcoma. Lesions of prolonged therapy displayed decreased cellularity and abundant new bone, resembling low-grade central osteosarcoma. However, Roitman et al. (70) failed to find a clear association between treatment length of denosumab and extent of histologic changes. They reviewed histologic slides of 9 patients receiving denosumab and revealed that cellular atypia or patterns of ossification was less frequent but more relevant. These histologic feathers resemble an undifferentiated pleomorphic sarcoma, a conventional osteosarcoma or a low-grade central osteosarcoma. The Pseudosarcomatous changes were also described by Santosh et al. (71). A pseudosarcomatous spindle cell proliferation with osteoid matrix might have been confused with osteosarcoma after 9 cycles of denosumab therapy. In another case report of GCTB in the distal ulna, Sanchez-Pareja et al. (72) presented that densely cellular foci with atypical cells and osteoid deposition mimicked high-grade osteosarcoma after 6 weeks of denosumab treatment. They emphasized the difficulty in histologic evaluation of GCTB early in the course of denosumab treatment.

What accounts for the dramatic histological changes caused by denosumab? It appears to reflect a shift in the balance from RANK-mediated osteoclastic bone resorption to bone formation induced by stromal cells after denosumab therapy. Under physiological conditions, it is osteoclasts that induce osteoblastic bone formation by various growth factors including TGF-β and IGF-1 in the context of bone remodeling (73). In GCTB, however, the osteoclasts probably suppress the osteogenic differentiation of stromal cells via various mediating factors (32, 74). After denosumab therapy, both osteoclast maturation from precursors and function are blocked due to the inhibition of RANK/RANKL axis. Therefore, new balance may be in favor of bone formation, allowing surviving stromal cells to undrape their osteogenic nature in the microenvironment free of osteoclasts. However, this shift does not reflect the terminal differentiation of stromal cells which has a preosteoblast phenotype since the morphology reverts to classic GCTB after withdrawal of denosumab treatment (23).

Mukaihara et al. (75) performed comparative proteomic analyses to explore molecular mechanisms underlying the therapeutic effect of denosumab. They identified five most dysregulated proteins, MMP9, LUM, KCRB, CAH2, PPA5. The first two were associated with the local aggressive behavior of GCTB (76, 77). The last three had crucial roles in osteoclast-mediated bone resorption (76, 78, 79). Kato et al. (80) revealed that all lesions treated by denosumab still contained plenty of G34W+ cells and harbored H3F3A mutations, indicating that neoplastic cells survived the denosumab therapy. Girolami et al. (81) displayed a significant reduction of microvessel density in GCTB after denosumab treatment although the underlying mechanism responsible for the antiangiogenic effect needed to be further expounded.

## How Long Should Denosumab Therapy be Continued?

Despite numerous trials involving denosumab for the treatment of GCTB, much about the optimal therapy duration remains unknown. There is a major concern that cessation of denosumab correlates with a higher rate of subsequent local recurrence (47). Palmerini et al. (82) revealed that after a median of 8 months with the range from 7 to 15 months of discontinuing denosumab, 40% of patients of GCTB had tumor progression. Matcuk et al. (83) presented a case report of rapid recurrence of GCTB in a woman aged 24 after the discontinuation of long-term denosumab therapy. The patient had good and sustained tumor control for over 2 years during denosumab therapy. However, only within 2 months of cessation, the tumor showed rapid recurrence and progression with growth. Worse still, it was resistant to reinitiating denosumab therapy, ultimately necessitating below-the-elbow amputation. The authors, therefore, recommended life-long denosumab therapy. In the largest clinical trial on GCTB using denosumab, Chawla et al. (12) reported that 26% (34/132) of surgically unsalvageable patients had disease recurrence or progression after cessation of denosumab. The authors recommended a reduced dose or less frequent administration of denosumab for maintenance in patients with unresectable GCTB. Therefore, it is critical and urgent to evaluate the risk of relapse following denosumab cessation in the prospective clinical trials. If the tumor recurrence is inevitable after drug withdraw, patients have to receive life-long treatment. Lingering unknowns on long term safety, the optimal maintenance dose and frequency schedule and therapeutic strategy for female patients of baby-bearing age remain to be explored.

## Conclusions

In the initial trials, Denosumab was deemed as an exciting, new targeted therapy option for patients with GCTB. The usage of neoadjuvant denosumab aims to facilitate surgery, making intralesional curettage or resection technically easier and feasible, thereby hoping for local tumor control. However, more and more studies displayed the negative effects of denosumab therapy on GCTB. (1) Denosumab selectively targeted osteoclastic cells but had limited inhibitory effect on neoplastic stromal cells, which persisted and remained proliferative on cessation of drug. (2) Denosumab may increase the risk of local recurrence in patients of GCTB treated with curettage. The thickened new bone induced by denosumab, in which tumor cells got trapped, made it difficult for surgeon to delineate the true extent of the tumor and curettage adequately. As a result, if curettage is feasible, we do not recommend using preoperative denosumab, unless the benefit outweighs the possibility of local recurrence. (3) Denosumab may cause the malignant transformation of GCTB. GCTB is a benign tumor in a young population and rarely life threatening even if lung metastasis occurs. However, despite low incidence rate and unknown mechanisms, sarcomatous transformation induced by denosumab has a poor outcome, which is a fatal blow to young patients. Based on these negative effects and unanswered questions regarding optimal use of denosumab, we recommended strongly application of this drug with caution for the treatment of GCTB, only when the burden of down-staging to perform a lesser morbid procedure outweighs the potential chance of local recurrence. Collaborative clinical trials and rigorous data collection are mandated to identify the optimum indications for using denosumab in GCTB and to ascertain the role that denosumab plays in malignant transformation and high recurrence risk.

## Author Contributions

HL wrote most of the manuscript. JG and YG made the figures and tables. MZ conceived and revised the manuscript. NL and ZY thoroughly revised and amended the manuscript. All authors contributed to the article and approved the submitted version.

## Conflict of Interest

The authors declare that the research was conducted in the absence of any commercial or financial relationships that could be construed as a potential conflict of interest.
